# Integrating real-time OUR monitoring with adaptive feeding for enhanced antibody production

**DOI:** 10.1186/s40643-025-00961-x

**Published:** 2025-12-20

**Authors:** Xin-Ran Zhang, Qingyuan Ran, Botao Zhang, Yong-Mei He, Liang Zhao, Wen-Song Tan, Qian Ye

**Affiliations:** 1 State Key Laboratory of Bioreactor Engineering, East China University of Science and Technology, 130 Meilong Road, Shanghai, P. O. Box 309#, 200237 China; 2 Shanghai Collaborative Innovation Center For Biomanufacturing Technology (SCIBT), Shanghai, P. O. Box 309#, 200237 China; 3 Shanghai BioEngine Sci-Tech CO., LTD, Shanghai, P. O. Box 309#, 201203 China; 4 Key Laboratory of Photoelectric Conversion and Utilization of Solar Energy, Qingdao New Energy Shandong Laboratory, Qingdao Institute of Bioenergy and Bioprocess Technology, Chinese Academy of Sciences, Shandong, Qingdao China

**Keywords:** Oxygen uptake rate (OUR), Fed-batch culture, Chinese hamster ovary (CHO) cells, Process analytical technologies (PAT), Continuous feeding strategy

## Abstract

**Background:**

Fed-batch culture is a well-established and widely adopted platform for industrial antibody production using Chinese Hamster Ovary (CHO) cells. However, conventional fixed feeding strategies often fall short in meeting the dynamically changing nutrient demands of cells, leading to metabolic imbalance and suboptimal productivity. Oxygen Uptake Rate (OUR), as a real-time indicator of cellular respiratory activity, is tightly coupled to nutrient metabolism and holds strong potential for guiding adaptive, demand-driven feeding strategies.

**Methods:**

To understand the decline in specific productivity (Q_P_) during the late stationary phase (LSP) under a conventional reference feeding (RF) strategy, we examined whether it stemmed from cell-intrinsic metabolic changes or from environmental stressors such as nutrient imbalance, by-product accumulation, and osmotic pressure. Based on these insights, we developed an OUR-based continuous feeding (OBCF) strategy and benchmarked its performance against the RF strategy. Mechanistic understanding was elucidated through metabolic flux and transcriptional analyses.

**Results:**

The RF strategy resulted in a mismatch between nutrient supply and cellular demand during LSP, triggering osmotic stress and limiting antibody expression. In contrast, the OBCF strategy dynamically aligned nutrient delivery with cellular respiration, thereby mitigating osmotic stress and reshaping intracellular metabolism. Notably, OBCF enhanced pyruvate utilization and TCA cycle activity, promoted amino acid catabolism, and suppressed by-product accumulation. These metabolic improvements led to a 52% increase in specific productivity (Q_P_) and a 32% increase in total antibody yield during LSP, along with reduced batch-to-batch variability.

**Graphical abstract:**

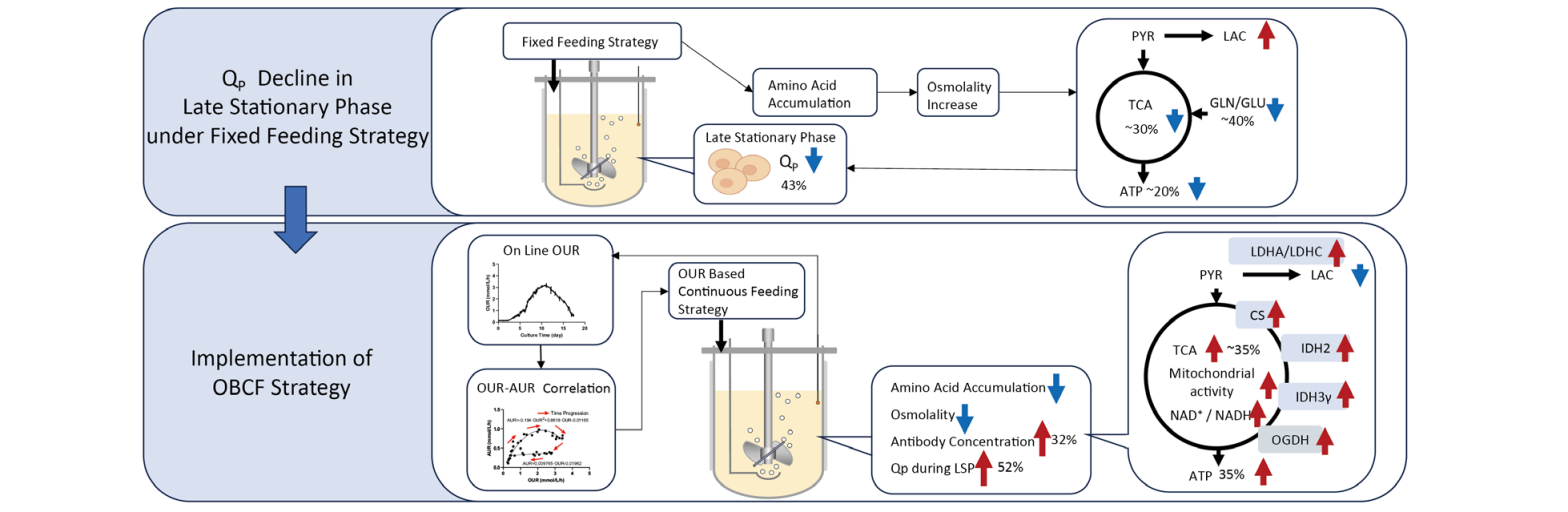

**Supplementary Information:**

The online version contains supplementary material available at 10.1186/s40643-025-00961-x.

## Introduction

Monoclonal antibodies have emerged as pivotal therapeutics for cancer and immune-related disorders, with global sales projected to exceed $346.6 billion by 2028 (Townsend et al. 2024). Among the available production systems, Chinese hamster ovary (CHO) cells cultured in fed-batch processes remain the industry standard, owing to their robust expression capacity, product quality, and scalability (Zhu et al. 2017). Despite the widespread adoption and industrial maturity of this platform, significant challenges persist, particularly in managing the delicate balance between cellular nutrient requirements, the accumulation of metabolic byproducts, and osmolality buildup, which compromise production efficiency and product consistency (Xu et al. 2023).

In industrial CHO fed-batch cultures, open-loop feeding strategies are commonly adopted for their operational simplicity and scalability. These methods typically follow predefined feeding to the real-time cellular metabolic state. Consequently, such static feeding schemes may fail to meet the dynamic nutrient demands of cells for antibody production, particularly during the later culture phases when cellular metabolism undergoes significant shifts. Although approaches such as design of experiments (DOE) and algorithm-guided medium optimization have been explored to enhance culture performance (Li et al. 2021; Zhou et al. 2023), these methods are inherently static and unable to accommodate the dynamic shifts in nutrient requirements throughout the culture process. These limitations underscore the pressing need for adaptive, real-time feeding strategies that can align nutrient supply with the evolving metabolic requirements of the cells throughout the production process.

To improve the adaptability of feeding strategies, several feedback control approaches have been developed based on measurable parameters such as pH (Gagnon et al. 2011; Hiller et al. 2017; Li et al. 2012), viable cell density (VCD) (Lu et al. 2013; Yu et al. 2011; Zhang et al. 2015), and nutrient concentrations (Yu et al. 2011). While these approaches provide partial feedback, they rely on indirect or lagging indicators, which may not accurately reflect cellular metabolic states. Raman spectroscopy has also been explored as a potential tool to monitor multiple nutrients simultaneously (Domján et al. 2020), however, its application is hindered by high instrumentation costs and the need for complex chemometric models, limiting its feasibility for routine real-time process control.

In contrast, the oxygen uptake rate (OUR) serves as a direct and sensitive indicator of metabolic activity in aerobic systems like CHO cell cultures. As a key readout of central carbon metabolism, including glycolysis and the tricarboxylic acid (TCA) cycle, OUR dynamically reflects fluctuations in cellular energy demand and biosynthetic activity. Previous studies have leveraged OUR to estimate concentrations of critical nutrients like glucose and glutamine and to guide nutrient supplementation strategies (Gálvez et al. 2012; Martínez-Monge et al. 2022; Zhou et al. 1995). However, approaches based on OUR feedback have mostly targeted individual nutrients, which do not fully reflect the complexity of cellular nutrient requirements. As amino acids collectively contribute to biomass synthesis, energy metabolism, and redox balance, a more integrated strategy is required to coordinate their supply.

In this study, we systematically characterized the dynamic changes in cellular physiology, nutrient consumption, and metabolic flux during CHO fed-batch culture under a conventional static feeding strategy, hereafter referred to as the reference feeding (RF) strategy. Our analysis revealed significant limitations of the RF strategy, most notably the accumulation of amino acids, which leads to increased osmolarity and a subsequent decline in specific productivity (Q_P_) during the late stationary phase. To overcome the mismatch between static feeding schedules and dynamic cellular metabolism, this study aimed to establish a real-time feedback-controlled feeding strategy based on cellular OUR. Building upon our previously developed OUR monitoring platform, we constructed a predictive model that quantitatively link OUR to the total amino acid uptake rate (AUR). This enabled, for the first time, dynamic control of comprehensive amino acid supplementation in CHO fed-batch processes. The resulting strategy, termed OUR-based continuous feeding (OBCF), was implemented through an online soft sensor and evaluated in bioreactor cultures in comparison with a conventional open-loop feeding scheme. Through integrated analyses of metabolic flux distribution, transcriptional profiles, and process performance, we demonstrated that the OBCF strategy effectively improves energy metabolism, stabilizes specific productivity, and enhances final yield and process robustness. These findings highlight the potential of OUR-driven control systems in enabling more adaptive and efficient bioprocesses for biotherapeutic production.

## Materials and methods

### On-line OUR monitoring

Real-time online monitoring of the OUR was conducted using the OUR soft sensor previously developed in our laboratory (Zhang et al. 2024) based on the stationary liquid phase balance principle. To overcome limitations of this principle when applied to aerated stirred-tank bioreactors, the method includes optimized estimation of saturation dissolved oxygen concentration (C*) and volumetric mass transfer coefficient (k_L_a). The monitoring program was implemented on the LabVIEW platform, enabling accurate, repeatable, and sensitive real-time OUR estimation without the need for additional hardware or manual intervention.

### Cell culture

#### Cell line and culture medium

A GS-CHO cell line expressing a monoclonal antibody (Junshi Biosciences, China) was used in this study. The cells were cultured in suspension under serum-free conditions and routinely expanded in CD CHO Fusion medium (Merck, USA), a chemically defined commercial basal medium.

The chemically defined basal medium Eden B400S and feeding media Eden F400aS and Eden F200bS (Bioengine, China), which were custom-designed for the specific CHO cell line following the formulation develop strategy described by Zou et al. (2020) were used for the fed-batch cultures.

#### Feeding strategy

For the reference feeding (RF) strategy, 3.0% (v/v) Eden F400a S and 0.3% Eden F200bS were added every 24 h starting at 72 h of cultivation, except at 96 h. For the OUR-based continuous feeding (OBCF) strategy, the feeding rate* F* (L/h) was calculated using the following equation:

$$ F = \frac{{AUR \times V}}{{C_{{15AA}} }} $$

where* V* refers to the culture volume (L); $\:{C}_{15AA}$ is the total concentration of 15 continuously consumed amino acids (Arg, Asn, Asp, Cys, His, Ile, Leu, Lys, Met, Phe, Ser, Thr, Trp, Tyr, Val) in the feed (mmol/L), and* AUR* (Amino Acid Uptake Rate) represents the uptake rate of the 15 amino acids (mmol/L/h).

The relationship between AUR and OUR was defined by two separate equations, depending on the trend of OUR. When OUR was increasing:

$$ AUR =  -\, 0.194 \cdot OUR^{2}  + 0.8618 \cdot OUR - 0.01165 $$

When OUR was decreasing:

$$ AUR = 0.009765 \cdot OUR - 0.01962 $$

In both RF and OBCF strategies, a 1 mol/L glucose stock solution was added daily to maintain the glucose concentration above 5 mmol/L.

#### Stirred tank bioreactor Fed-batch processes

Exponentially growing seed cells were inoculated into a sterile 1 L bioreactor (Applikon, Netherlands) equipped with calibrated pH and dissolved oxygen electrodes. Cultures were initiated at a working volume of 500 mL and a seeding density of 5 × 10^5^ cells/mL. The bioreactor was aerated at 0.1 vvm using air to maintain positive pressure. Dissolved oxygen was controlled at 40% through oxygen sparging, while pH was maintained at 7.0 ± 0.1 using 1 mol/L NaOH and carbon dioxide sparging. The culture was held at 37 °C with a stirring speed of 200 rpm.

#### Shake tubes Fed-Batch processes

To investigate the cause of Q_P_ decline during the late stationary phase (LSP) of fed-batch culture, a series of experiments were conducted in shake tubes (50 mL TubeSpin^®^ bioreactors, TPP, Switzerland). Exponential growing seed cells were inoculated at 5 × 10^5^ cells/mL into 20 mL working volumes. Cultures were maintained at 220 rpm, 37 °C, 5% CO_2_, and 85% humidity in a shaker incubator (Kuhner, Switzerland). Feeding followed the RF strategy described previously. The cells were subjected to the following treatments:

1. Supernatant exchange test: Group 2 was inoculated three days after Group 1. On Day 10 (Group 1) and Day 7 (Group 2), cultures were centrifuged (1000 rpm, 5 min). The supernatant from Group 2 was used to resuspend the cells from Group 1 (named D7 cell + D10 S), and vice versa (named D10 cell + D7 S). The cultures were then continued until cell death occurred.
2. Dose-response to feeding volume (Late course): From Day 9 onward, the feeding volume of F400aS for each bolus was adjusted to 1.5% (v/v), 2.0% (v/v), 2.5% (v/v), 3.0% (v/v), 3.5% (v/v), 4.0% (v/v), and 4.5% (v/v), respectively. Volume of F200bS was one-tenth that of F400aS.
3. Medium replacement test: On Day 10, cells were centrifuged and resuspended in either low-osmolarity basal medium (B400S, Low-osmotic Media Replacement, LMR) or B400S supplemented with 9.0% F400aS to match the Day 10 osmolarity (Isotonic Media Replacement, IMR).
4. Dose-response to feeding volume (Full Course): The feed volume of F400aS for each bolus was adjusted to 1.5%, 2.0%, 2.5%, 3.0%, 3.5%, 4.0%, and 4.5% (v/v). The volume of F200bS is one-tenth that of F400aS.
5. Osmolality stress test: Osmolality was raised by 25 or 45 mOsm/kg at Day 9 by adding 1 mol/L NaCl. For Day 4, only a 25 mOsm/kg increase was applied, which gradually reached ~ 46 mOsm/kg by Day 9 due to continuous feeding; thus, no separate Day 4 45 mOsm/kg group was included.

The experimental setups for Sect. “Shake tubes fed-batch processes” (items 1–4) are illustrated in Supplementary Figure S1, while the osmolality stress test in the same section (item 5) is shown in Fig. 3G.

### Phase-Specific µ and Q_P_ calculation

For each culture phase (e.g., ESP, LSP), the µ and Q_P_ were determined as the mean of their daily values within the phase. Error bars represent the standard deviation of daily values across the corresponding phase.

### Off-line analytical methods

Cell density, viability, and diameter were measured using a Countstar^®^ automated cell counter (Ruiyu, China) based on the trypan blue exclusion method. Glucose, lactate, and ammonia concentrations were determined using glucose assay kits, L-lactate test kits, and blood ammonia assay kits, respectively (Jiancheng Bioengineering Institute, China). Antibody concentrations in the culture supernatant were detected using a Poros A affinity chromatographic column (Thermo Scientific, US) and an HPLC system (Agilent, US). Amino acid concentrations in the supernatant were analyzed using pre-column derivatization HPLC with a YORBAX Eclipse Plus C18 column (4.6 × 150 mm, 3.5 μm, Agilent, US). Mitochondrial membrane potential and NAD^+^/NADH assay kits were sourced from Beyotime (China).

### Metabolic flux analysis

Metabolic Flux Analysis (MFA) was performed as previously described (Zhang et al. 2016). Essential metabolic pathways included glycolysis, the tricarboxylic acid (TCA) cycle, biomass synthesis, antibody production, citrate pyruvate cycle, and amino acid metabolism, encompassing 32 intracellular metabolites and 29 biochemical reactions (detailed in Supplementary Table S1). Samples were collected on cultivation days 2, 5, 9, 12, and 15, and metabolite concentrations were measured using HPLC. Data were processed using an Excel-based metabolic flux analysis model developed by Mr. Jongchan Lee (Gambhir et al. 2003), which applied linear algebra techniques under the assumption of a respiratory quotient (RQ) of 1.

### Principal component analysis

Principal component analysis (PCA) was performed using Python to compare the central carbon metabolism fluxes of the two experimental groups (RF strategy and OBCF strategy) across five phases of fed-batch culture. The metabolic flux data were standardized using the StandardScaler to ensure comparability. PCA was applied to reduce the high-dimensional flux data to a single principal component, capturing the major variance in the data. The Euclidean distance between the centroids of the two groups in the PCA space was calculated for each phase, providing a quantitative measure of metabolic differences between the two feeding strategies.

### Analysis of enzyme transcription levels

Quantitative Real-Time PCR (qPCR) was utilized to assess the transcription levels of key enzymes in central carbon metabolism. RNA was extracted from cell samples using TRNzol Universal reagent (TIANGEN, China) and reverse-transcribed into cDNA with the FastKing cDNA First Strand Synthesis Kit (TIANGEN, China). qPCR was conducted using specific primers (Supplementary Table S2) designed to span exon-exon junctions, together with Hieff UNICON^®^ Advanced qPCR SYBR Master Mix (YEASEN, China), on an Applied Biosystems qRT-PCR system (USA). The thermal cycling conditions included an initial denaturation at 95 °C for 2 min, followed by 35 cycles of 95 °C for 10 s and 60 °C for 30 s. Each sample was technically replicated three times, and gene expression normalization was performed using β-actin as an internal control, confirmed for stability under experimental conditions. Relative expression levels were calculated using the 2^−ΔΔCt^ method.

### Specific ATP production rate

The specific ATP production rate (Q_ATP_) was defined as the estimated rate of ATP generation from central carbon metabolism, normalized to viable cell concentration, and was calculated using the following formula:

$$   \begin{aligned}   Q_{{ATP}}  &  = \frac{{J_{{GLC - PYR}} }}{6} \times 2 + \frac{{J_{{OAA - AKG}} }}{5} \times 2.5 \\     & \quad  + \frac{{J_{{AKG - SUC}} }}{4} \times 2.5 + \frac{{J_{{SUC - FUM}} }}{4} \times 1.5 \\     & \quad  + \frac{{J_{{MAL - OAA}} }}{4} \times 2.5 + \frac{{J_{{PYR - ACC}} }}{2} \times 2.5 \\     & \quad  + \left( {\frac{{J_{{GLC - PYR}} }}{6} \times 2 - \frac{{J_{{PYR - LAC}} }}{3}} \right) \times 2.5 \\     & \quad  + \frac{{J_{{SUC - FUM}} }}{4} \\  \end{aligned}  $$

In this equation,* J* represents the metabolic fluxes of specific reactions involved in central carbon metabolism (mmol C/10^9^ cells/h). For clarity, metabolite abbreviations used in the formula are as follows: GLC for glucose, PYR for pyruvate, LAC for lactate, OAA for oxaloacetate, AKG for α-ketoglutarate, SUC for succinate, FUM for fumarate, MAL for malate, and ACC for acetyl-CoA, where each term corresponds to a different segment of the metabolic network.

### Statistical analysis

All data were analyzed using GraphPad Prism 10 (MathWorks, US), and statistical comparisons were performed with a two-tailed Student’s t-test. Statistical significance was defined as *p < 0.05, **p < 0.01, ***p < 0.001, and ****p < 0.0001, indicating varying degrees of significance.

## Results

### Identification of Q_P_ reduction in the late stationary phase of Fed-Batch culture

To investigate the dynamics of CHO cell productivity and identify critical factors limiting antibody yield, we first evaluated a conventional open-loop reference feeding (RF) strategy in a 1 L stirred-tank bioreactor. This strategy involves fixed daily additions of concentrated medium without real-time feedback.

**Fig. 1:**
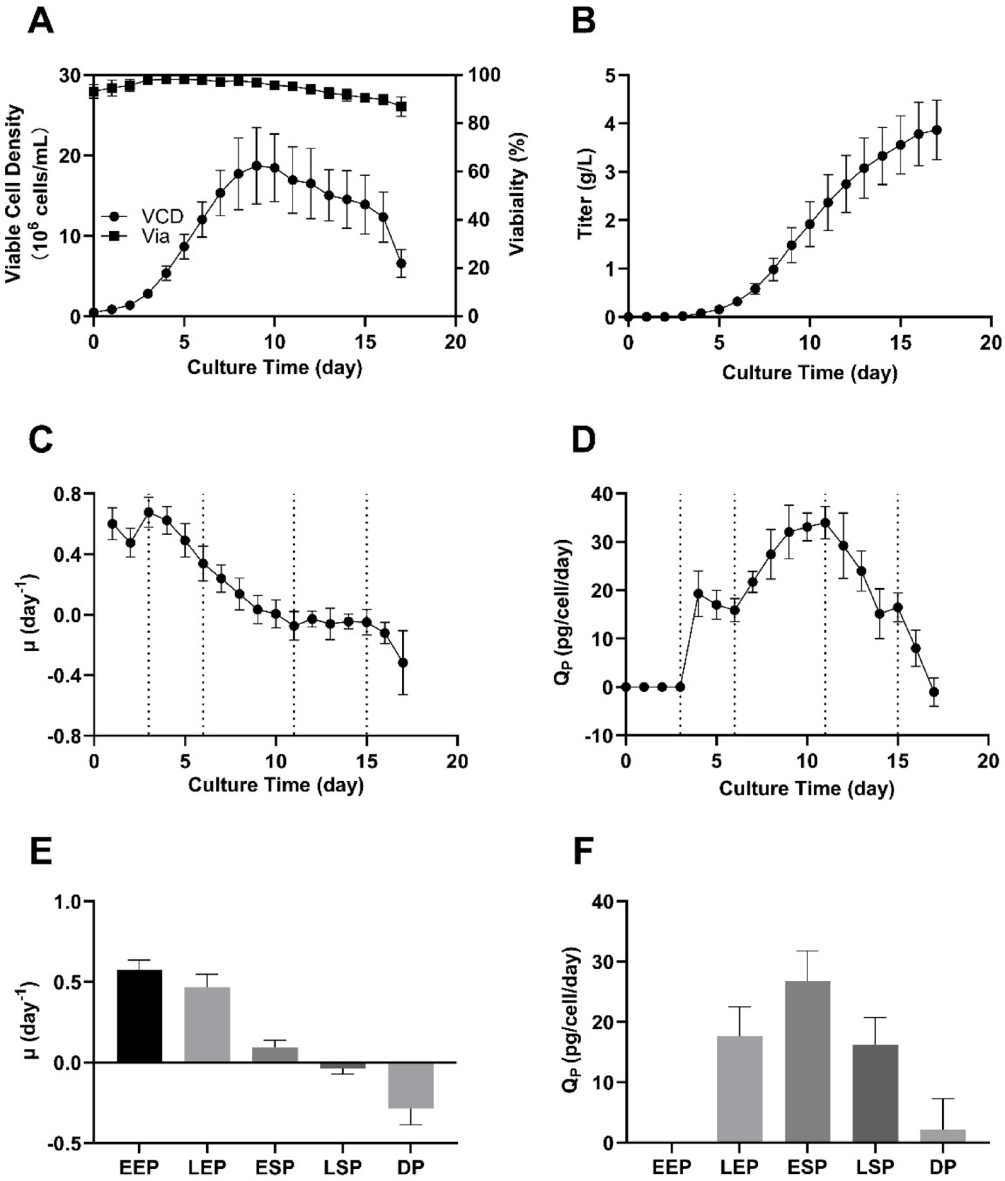
CHO fed-batch culture using the reference feeding (RF) strategy:** A** Viable cell density and viability, **B** Titer,** C** µ (Specific growth rate),** D** Q_P_ (Specific Antibody Production Rate), **E** average µ during the five phases of fed-batch culture,** F** average Q_P_ during the five phases of fed-batch culture

As shown in Fig. 1A and B, the culture reached a maximum viable cell density of 16.84 ± 2.15 × 10^6^ cells/mL and a final antibody titer of 3.81 ± 0.35 g/L on day 17. The temporal profiles of specific growth rate (µ) and specific antibody production rate (Q_P_) are presented in Fig. 1C and D. During the exponential phase, µ peaked at approximately 0.54 ± 0.09 day^-1^. It declined to 0.03 ± 0.07 day^-1^ in the stationary phase and further dropped to -0.29 ± 0.10 day^-1^ in the decline phase.

Based on the onset and dynamics of cellular growth and antibody production, the fed-batch process was divided into five distinct phases: early exponential phase (EEP, D0-D3), late exponential phase (LEP, D3-D6), early stationary phase (ESP, D6-D11), late stationary phase (LSP, D11-D15), and decline phase (DP, D15-D17). The phase-specific profiles of µ and Q_P_ are summarized in Fig. 1E and F.

Notably, Q_P_ was not maintained throughout the stationary phase, it decreased from 26.77 ± 5.01 pg/cell/day in the ESP to 16.22 ± 4.54 pg/cell/day in the LSP, representing a 40% reduction. As most antibody accumulation occurs during the stationary phase, this Q_P_ drop substantially limited overall productivity. A simple extrapolation suggests that maintaining Q_P_ at ESP levels during the LSP could have increased the final titer by approximately 0.86 g/L.

These findings highlight a key limitation of the RF strategy: the inability to sustain high Q_P_ in later culture phases. To better understand the potential causes of this decline, we further investigated the metabolic characteristics of CHO cells during the ESP and the LSP, with a particular focus on intracellular metabolic flux distribution and energy metabolism, given their close relationship with antibody production.

### Intracellular metabolic changes associated with Q_P_ decline

**Fig. 2:**
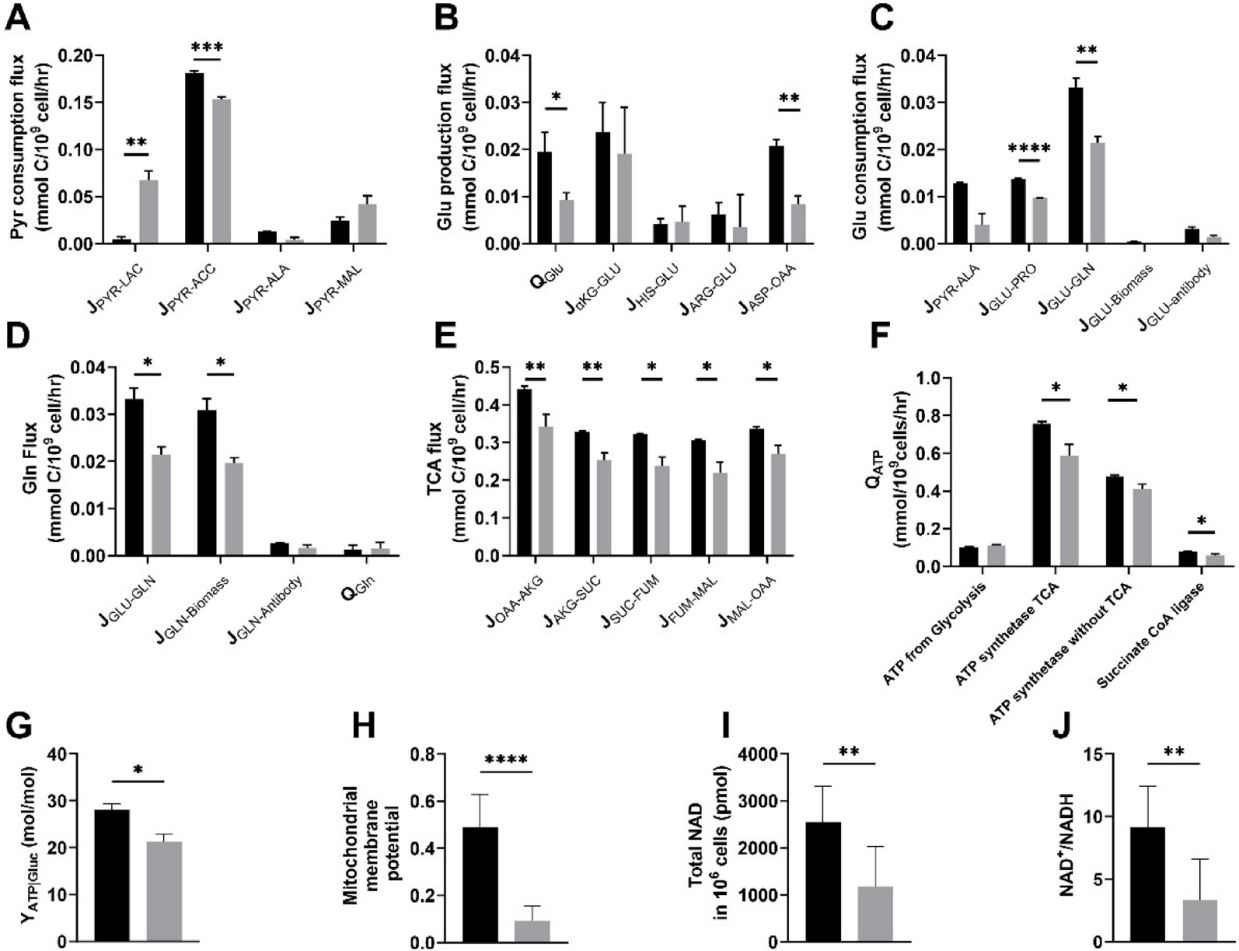
Key metabolic fluxes and energy metabolism in the early stationary phase (ESP, black) and the late stationary phase (LSP, grey) of the fed-batch culture with RF strategy. **A** Pyruvate consumption flux,** B** Glutamic acid production flux,** C** Glutamic acid consumption flux, **D** Glutamine flux, **E** TCA flux, **F** specific ATP production rate (Q_ATP_), **G** ATP yield per mole of glucose (Y_ATP|Gluc_), **H** Mitochondrial membrane potential (Normalized JC-1 red/green fluorescence intensity),** I** Total intracellular NAD levels,** J** NAD^+^/NADH ratio

To elucidate the cellular basis of the Q_P_ reduction observed during LSP, intracellular metabolic fluxes and energy metabolism were analyzed. The detailed flux values are provided in Supplementary Table S3, and key transitions in metabolic and energy-related pathways are illustrated in Fig. 2.

While glucose uptake and glycolytic fluxes remained relatively constant (Supplementary Table S3), the destination of pyruvate showed substantial changes from ESP to LSP: the fraction of pyruvate entering the TCA cycle (J_PYR−ACC_) decreased from 91% to 76%, whereas the proportion diverted to lactate production (J_PYR−LAC_) increased from 2.0% to 22% (Fig. 2A). This shift from oxidative phosphorylation toward increased lactate production may reduce ATP generation efficiency, thereby contributing to the decline in Q_P_.

For the GS-CHO cells used in this study, both glutamine and glutamate serve as major nitrogen and carbon sources, contributing to biosynthetic processes and energy metabolism. During LSP, the overall flux of glutamate decreased significantly, primarily due to reduced glutamate uptake and lower transamination flux from Aspartate to oxaloacetate(J_ASP-OAA_), which contributes to glutamate formation via the transamination of aspartate with α-ketoglutarate (Asp + α-KG→OAA + Glu) (Fig. 2B). Additionally, the fluxes associated with glutamate utilization for proline biosynthesis (J_GLU-PRO_) and glutamine biosynthesis (J_GLU-GLN_) also declined notably (Fig. 2C). For glutamine, although the total glutamine flux was reduced, its relative distribution across pathways remained unchanged (Fig. 2D). These results indicate that glutamate metabolism becomes notably suppressed during LSP, which may partially account for the reduced biosynthetic capacity and Q_P_ observed in this phase.

Fluxes through various steps of the TCA cycle reactions declined by 23%-36% during the transition from ESP to LSP (Fig. 2E). ATP production via glycolysis remained unchanged; however, ATP synthesis via ATP synthase decreased significantly. The total Q_ATP_ in LSP was 1.16 ± 0.02 mmol/10^9^ cells/h, which is approximately 20% lower than the 1.42 ± 0.00 mmol/10^9^ cells/h observed in ESP (Fig. 2F). Furthermore, the ATP yield per mole of glucose (Y_ATP|Gluc_) declined from 28.16 ± 0.79 mol/mol in ESP to 21.24 ± 1.16 mol/mol in LSP (Fig. 2G). Indicators of mitochondrial function and energy metabolism, including mitochondrial membrane potential, total intracellular NAD levels, and the NAD⁺/NADH ratio also exhibited significant decreases in LSP (Fig. 2H-J). These results suggest that energy metabolism became increasingly inefficient during LSP, characterized by impaired mitochondrial function and reduced oxidative phosphorylation. Given that antibody production is a highly energy-intensive process, such declines in ATP synthesis capacity may directly limit biosynthetic efficiency and contribute to the observed reduction in Q_P_.

To further investigate the molecular basis of these metabolic changes, the transcriptional levels of key enzymes involved in central carbon metabolism were analyzed (Supplementary Figure S2 and S3). Notably, the *LDHA/LDHC* transcript ratio was significantly elevated in the LSP, indicating a shift toward LDH isoenzymes that favor lactate production over its oxidation, consistent with increased lactate formation. Meanwhile, the markedly reduced expression of *IDH2*, an essential TCA cycle enzyme catalyzing isocitrate to α-ketoglutarate, likely contributes to the suppressed TCA cycle activity during LSP.

Collectively, these findings reveal a pronounced shift in cellular energy metabolism and mitochondrial function during LSP. This metabolic reprogramming is likely influenced by extracellular conditions, prompting further investigation into how the culture environment contributes to the observed decline in productivity.

### Extracellular factors associated with Q_P_ decline

**Fig. 3:**
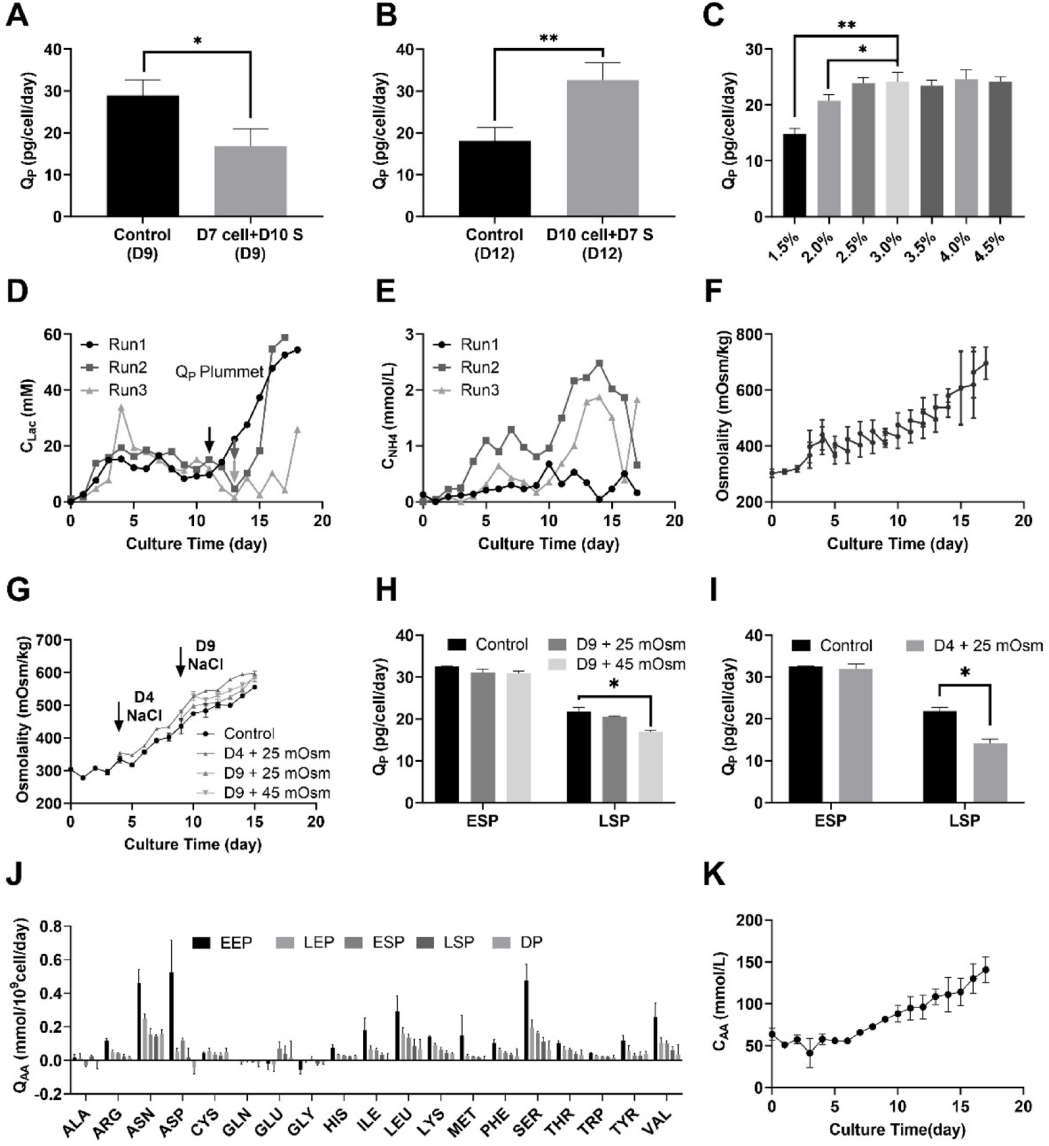
Analysis of factors contributing to the decline in Q_P_ during the LSP of fed-batch culture with RF strategy:** A**,** B** Changes in Q_P_ after exchanging the supernatant,** C** Effects of adjusting daily feed volume from day 9 on Q_P_,** D** Time-course profiles of lactate concentration during fed-batch culture, **E** Time-course profiles of ammonia concentration during fed-batch culture,** F** Osmolality before and after feeding during fed-batch culture, **G** Pre-feeding osmolality in osmotic regulation experiments, **H** Changes in Q_P_ following osmolality increase with NaCl starting on Day 9, **I** Changes in Q_P_ following osmolality increase with NaCl starting on Day 4,** J** specific amino acid consumption rate (Q_AA_) of five phases during fed-batch culture, **K** Time-course profiles of amino acid concentration during fed-batch culture

To investigate the environmental factors contributing to the Q_P_ reduction during the late stationary phase (LSP), we first sought to determine whether the decline was stemmed from intrinsic changes in cellular metabolism or extrinsic alterations in the culture environment. A supernatant exchange experiment was conducted, as detailed in Sect. “Shake tubes fed-batch processes”, to decouple these factors. As illustrated in Fig. 3A and B, two days post supernatant exchange, Q_P_ in the D7 cell cultured in D10 supernatant (D7 cell + D10 S) was dropped significantly to 16.85 ± 3.40 pg/cell/day, compared to the control (28.90 ± 3.07 pg/cell/day, *p* < 0.05). Conversely, Q_P_ of the D10 cells cultured in D7 supernatant (D10 cell + D7 S) increased significantly to 32.58 ± 3.39 pg/cell/day, relative to the control (18.19 ± 2.52 pg/cell/day, *p* < 0.01). These findings suggest that the Q_P_ decline during LSP is primarily driven by changes in the extracellular environment rather than by irreversible intracellular alterations.

To identify the specific environmental factors involved, several variables were examined, including process control parameters (temperature, pH, dissolved oxygen), nutrient availability, byproduct accumulation, and osmolality. Throughout the fed-batch process, process parameters were maintained within tightly controlled ranges, and neither glucose nor amino acids were depleted, except for the non-essential amino acid asparagine. (data not shown). These observations indicate that neither process parameters nor limitations in the main carbon and nitrogen sources was responsible for the observed Q_P_ reduction.

To evaluate whether nutrient overfeeding or underfeeding contributed to LSP Q_P_ decline, the daily feed volume of concentrated medium was adjusted from day 9 onward (prior to the onset of Q_P_ reduction), as described in Sect. “Shake tubes fed-batch processes”. As shown in Fig. 3C, feed rate modulation fails to alleviate, and may even exacerbate, the Q_P_ drop. For example, modifying the original 3% daily feed volume to 1.5% or 2.0% resulted in 39% and 14% reduction in Q_P_, respectively. These results indicate that the mismatched feeding volume did not account for the loss of productivity.

The potential inhibitory effects of metabolic byproducts, such as lactate and ammonia, were also evaluated (Fig. 3D-E). Although lactate accumulated during LSP, its concentration remained below 10 mM when Q_P_ began to decline, suggesting a non-causal association. Similarly, ammonia concentrations stayed under 2.5 mM throughout, a level generally considered non-inhibitory for CHO cells (Ha et al. 2022; Synoground et al. 2021). Thus, neither lactate nor ammonia appeared to be responsible for the Q_P_ reduction.

Collectively, these results suggest that process control conditions, primary nutrient availability, and accumulation of typical metabolic byproducts are unlikely to be the dominant contributors to the reduced Q_P_ observed during the late stationary phase.

The culture was conducted in a conventional fed-batch mode without medium removal. During this process, the continuous addition of concentrated feed and the accumulation of metabolic by-products led to a progressive increase in osmolality, as shown in Fig. 3F. Notably, the sharp decline in Q_P_ occurred on day 11, when the post-feeding osmolality reached 496 ± 22 mOsm/kg, approximately 25–45 mOsm/kg higher than on day 9. To investigate whether the increase in osmolality contributed to the Q_P_ decrease observed during the LSP, the culture medium osmolality was artificially elevated using a NaCl concentrate, as described in Sect. “Shake tubes fed-batch processes”.

Figure 3G shows the osmolality profiles over time in the Day 9 and Day 4 osmotic regulation experiments. As shown in Fig. 3H, During the LSP, osmolality showed a dose-dependent effect on Q_P_. A 45 mOsm/kg increase significantly reduced Q_P_ to 16.9 ± 0.3 pg/cell/day versus 21.8 ± 0.6 in the control, while a 25 mOsm/kg increase caused only a slight, non-significant decline, confirming osmolality as a contributing factor to productivity loss. To determine whether this effect stemmed from cumulative stress rather than acute osmotic shock, osmolality was increased earlier in the culture (day 4). As shown in Fig. 3I, although Q_P_ during ESP remained unaffected, a notable reduction was observed during the LSP. These results suggest that NaCl addition per se did not exert a direct inhibitory effect on Q_P_; instead, the progressive accumulation of osmolality—especially beyond a certain threshold—ultimately contributed to Q_P_ reduction.

To explore the origin of this osmotic buildup, specific amino acid consumption rates (Q_AA_) were analyzed across five culture phases (Fig. 3J). Q_AA_ gradually declined for 15 amino acids (Arg, Asn, Asp, Cys, His, Ile, Leu, Lys, Met, Phe, Ser, Thr, Trp, Tyr, Val) continuously supplied through feeding. As daily feed volume remained fixed, unmetabolized amino acids accumulated, leading to elevated AA concentrations up to 53.73 mM by day 11 and 99.36 mM by the end of culture (Fig. 3K). This accumulation alone could account for a 53–99 mOsm/kg rise in osmolality, implicating excess amino acids as major drivers of osmotic stress.

Taken together, these findings indicate that the Q_P_ decline during the LSP under the RF strategy was not due to nutrient limitation or toxic byproduct accumulation, but rather to amino acid accumulation and the resulting osmotic stress, reflecting a mismatch between nutrient supply and cellular uptake. Nonetheless, the comparison between LMR and IMR (Supplementary Figure S4) suggests that osmolality alone cannot fully account for the productivity loss, implying the involvement of additional inhibitory factors in the culture environment.

This extracellular disturbance may partially account for the intracellular metabolic reprogramming observed in Sect. “Intracellular metabolic changes associated with Q_P_ decline”, including TCA cycle suppression, impaired mitochondrial function, and reduced ATP synthesis capacity—all of which are critical for supporting high antibody productivity.

Even when fixed feeding volumes of 2.5% or 3.5% were applied from Day 3 onward, Q_P_ still declined during the LSP (Supplementary Figure S5), underscoring the limitations of static feeding approaches.

These findings highlight the importance for a favorable extracellular environment that dynamically adapts to cellular metabolic demands. To this end, the following section introduces an oxygen uptake rate (OUR)-based feeding strategy designed to sustain Q_P_ during the later stages of culture.

### Establishment and effect of OUR-Based feeding strategy

Given that oxygen uptake rate (OUR) reflects real-time cellular metabolic activity, it was employed as a feedback signal to dynamically adjust feeding volume. This OUR-based continuous feeding (OBCF) strategy aims to align nutrient supply with actual cellular demand and minimize osmotic stress caused by amino acid accumulation. The following section presents the rationale, implementation, and performance of the OBCF strategy.

**Fig. 4:**
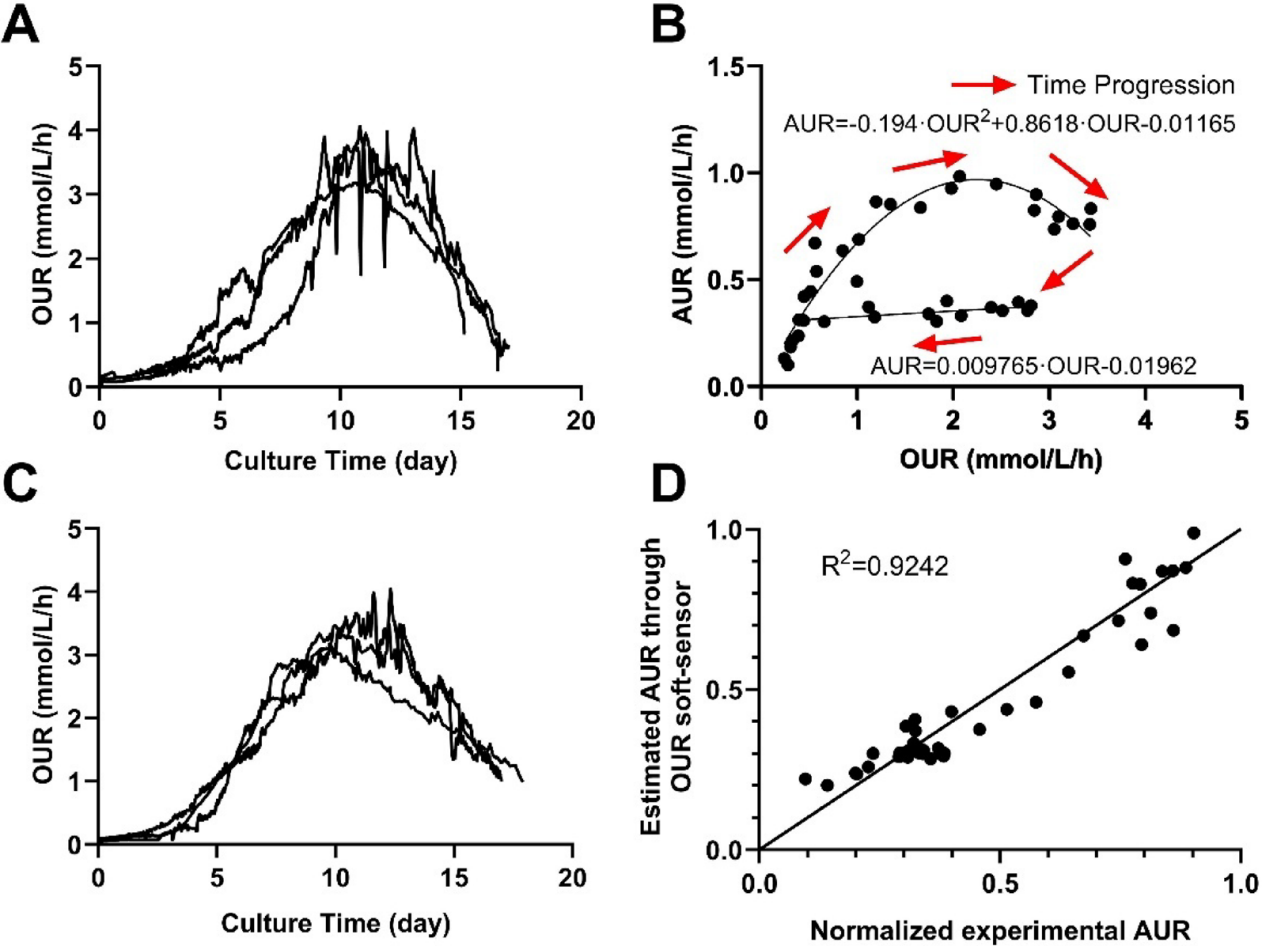
**A** Time profile of OUR in fed-batch culture using the RF strategy, **B** Relationship between OUR and the uptake rate of 15 continuously consumed amino acids (AUR), **C** Time profile of OUR in fed-batch culture using the OBCF strategy, **D** Comparison between the measured AUR and the AUR estimated by OUR

To implement the OBCF strategy, the relationship between OUR and the amino acid uptake rate (AUR) of 15 continuously consumed amino acids was first established under the RF strategy. As shown in Fig. 4A, OUR initially increases and then declined, peaking around day 11. The relationship between OUR and the AUR is depicted in Fig. 4B, with red arrows indicating the time progression. Two empirical functions were used to describe this relationship: a quadratic model during the rising phase of OUR and a linear model during the declining phase, as detailed in Sect. “Feeding strategy”. Based on these models, the feed rate (F, in L/h) was calculated using the equation provided in the same section, ensuring precise adjustment of amino acid supply to match cellular demand and avoid nutrient imbalance.

The effectiveness of the OBCF strategy was validated in three independent fed-batch cultivations, where feeding rates were dynamically regulated in real time based on OUR. As shown in Fig. 4C, the OUR profile remained consistent across replicates. Moreover, a strong linear correlation (R² = 0.9242) between the estimated and experimentally measured AUR (Fig. 4D)confirmed the high predictive accuracy of the OUR-based soft sensor for real-time estimation of amino acid requirement.

**Fig. 5:**
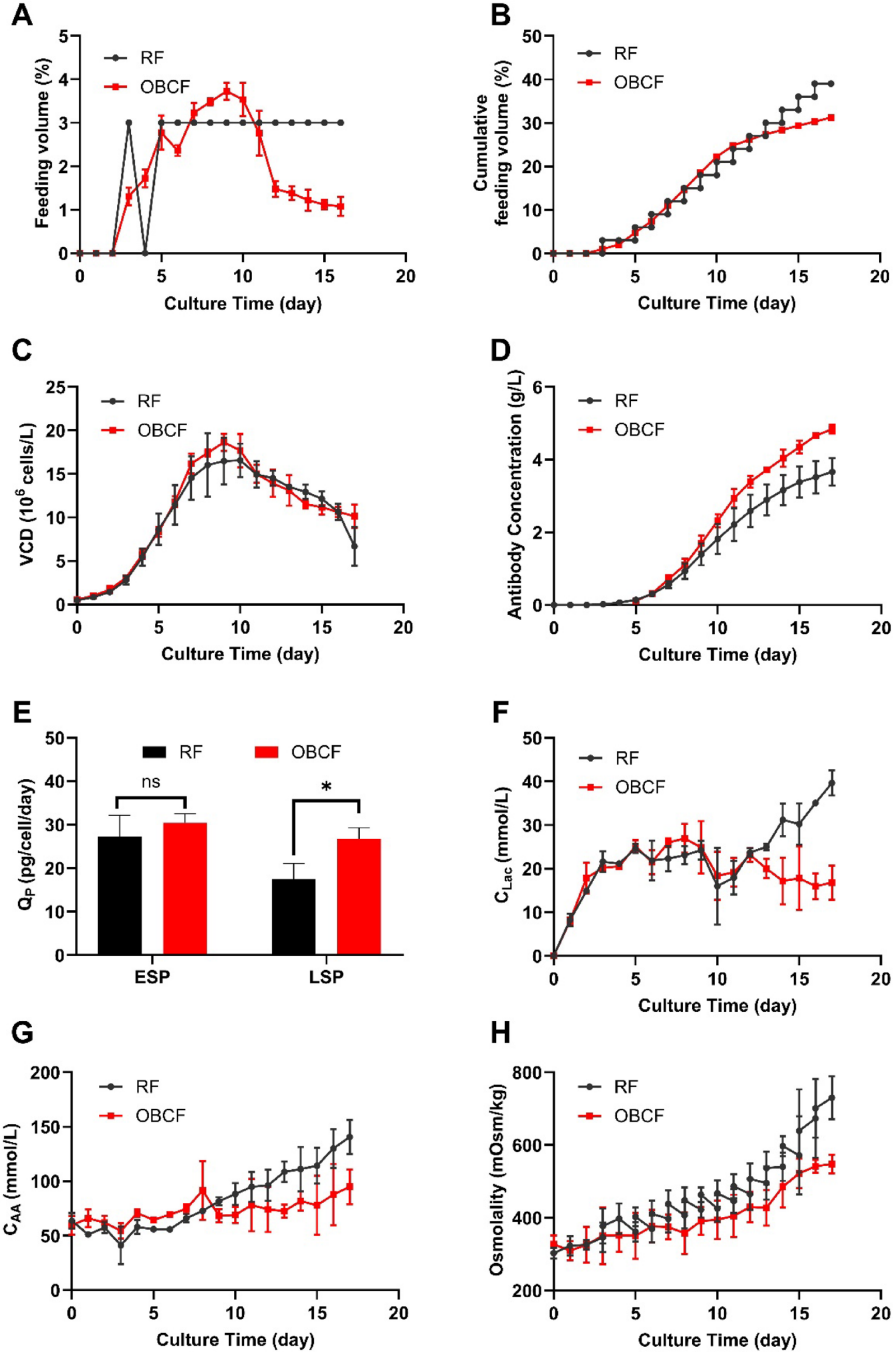
Comparison between fed-batch using reference feeding strategy (RF, black) and OUR-based continuous feeding strategy (OBCF, red):** A** Daily feeding volume (v/v), **B** Cumulative feeding volume (v/v), **C** Viable cell density, **D** Titer; **E** Q_P_ during ESP and LSP, **F** Lactate concentration, **G** Concentration of 15 continuously consumed amino acids, **H** Osmolarity

To evaluate the impact of the OBCF strategy on process performance, fed-batch cultures using the RF and OBCF strategies were compared (Fig. 5). Unlike the fixed-volume feeding in RF, the OBCF approach dynamically adjusted feed rates based on cellular demand. As shown in Fig. 5A and B, OBCF applied lower feed volumes during early culture, increased between days 7–10, and decreased after day 11, effectively avoiding nutrient oversupply in late stages.

Although maximum viable cell density remained similar (Fig. 5C), the final antibody titer under OBCF increased by 32% (Fig. 5D). This improvement was largely due to a 52% higher Q_P_ during the late stationary phase, whereas Q_P_ in the early stationary phase remained comparable between groups (Fig. 5E).

The OBCF strategy also alleviated byproduct accumulation. Lactate levels were comparable before day 12, but subsequently increased under RF, reaching 39.7 ± 2.0 mmol/L, while decreasing to 16.8 ± 3.2 mmol/L under OBCF (Fig. 5F). Amino acid accumulation was similarly reduced, with final concentrations at 94.9 ± 11.3 mmol/L under OBCF versus 140.5 ± 11.0 mmol/L under RF (Fig. 5G).

Correspondingly, osmotic stress was mitigated. Under RF, osmolality exceeded 530 mOsm/kg by day 13 and reached 730 ± 51 mOsm/kg by day 17, while OBCF maintained lower and more stable osmotic levels, ending at 574 ± 18 mOsm/kg (Fig. 5H).

**Fig. 6:**
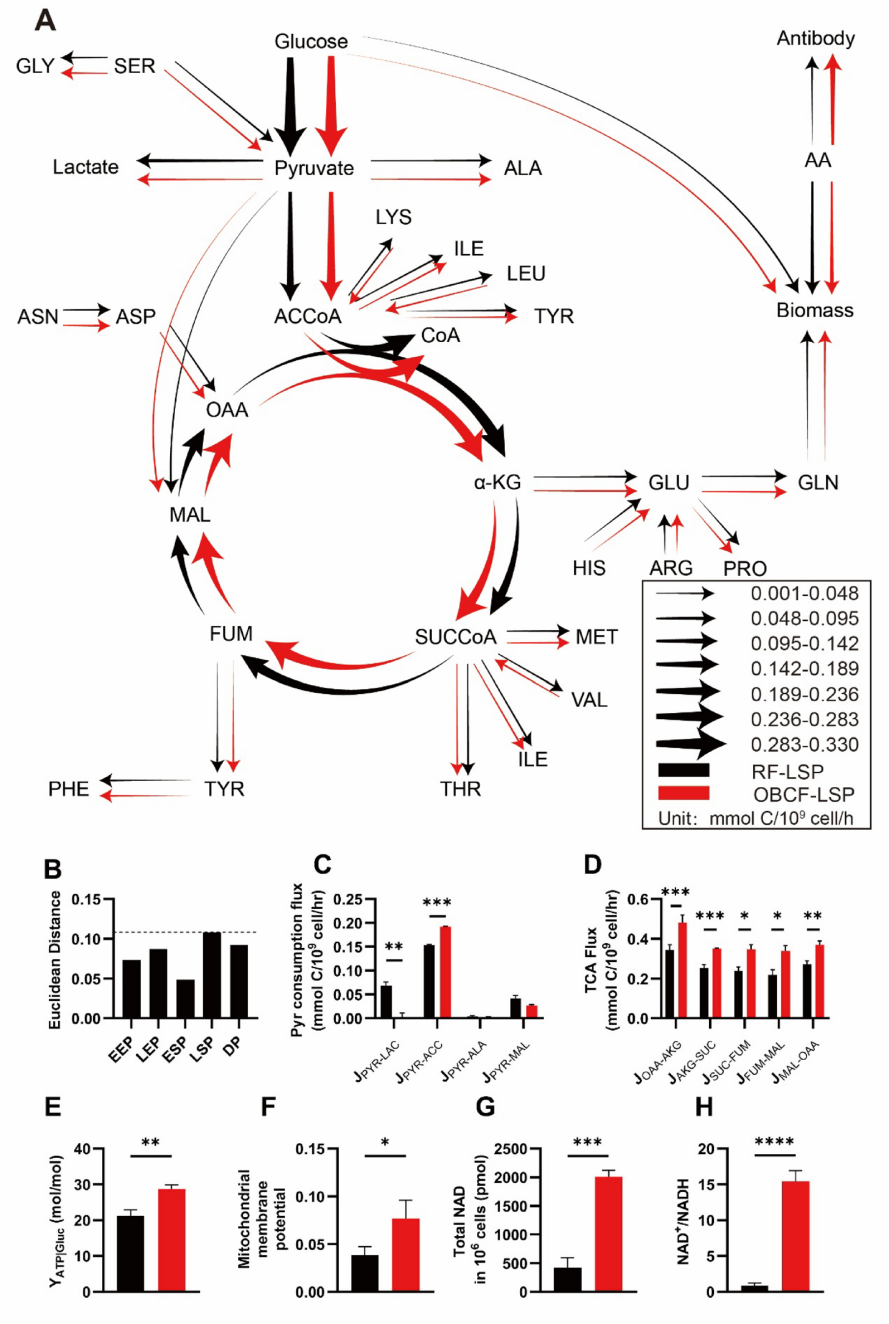
Intracellular metabolic characteristics under RF and OBCF strategies during the late stationary phase: **A** Intracellular flux map under RF (black) and OBCF (red) strategies, **B** PCA-based Euclidean distances of metabolic flux profiles across five culture phases, **C** Pyruvate consumption fluxes, **D** TCA cycle fluxes, **E** ATP yield per mole of glucose, **F** Mitochondrial membrane potential (JC-1 red/green ratio), **G** Total intracellular NAD levels, **H** NAD⁺/NADH ratio. Standard amino acids are abbreviated as GLY (glycine), SER (serine), ALA (alanine), LYS (lysine), ILE (isoleucine), LEU (leucine), TYR (tyrosine), PHE (phenylalanine), THR (threonine), VAL (valine), MET (methionine), GLU (glutamate), GLN (glutamine), HIS (histidine), ARG (arginine), PRO (proline), and AA (amino acids); central carbon metabolism intermediates are abbreviated as ACCoA (acetyl-CoA), CoA (coenzyme A), OAA (oxaloacetate), MAL (malate), FUM (fumarate), SUCCoA (succinyl-CoA), and α-KG (α-ketoglutarate)

To investigate the intracellular mechanisms underlying the enhanced productivity, metabolic fluxes were analyzed across the five culture phases. A comparative overview of flux maps comparing the RF and OBCF strategies is provided in Supplementary Figure S6. Principal component analysis (PCA) of the normalized flux data (Fig. 6B) revealed that the greatest divergence during the LSP, coinciding with the phase where Q_P_ improvements were most pronounced.

Comparative analysis of the LSP revealed that, during the OBCF process, the flux of pyruvate towards lactate (J_PYR−LAC_) was significantly reduced, whereas the flux of pyruvate entering the TCA cycle (J_PYR−ACC_) was markedly increased. Consequently, the proportion of pyruvate directed into the TCA cycle rose from 56% to 86% (Fig. 6C). This shift was accompanied by a 36–54% increase in TCA cycle flux across various phases of the OBCF process (Fig. 6D).

These metabolic changes improved cellular energy output. The ATP yield of glucose increased from 21.24 ± 1.16 to 28.6 ± 1.19 mol/mol under the OBCF strategy (Fig. 6E). Furthermore, mitochondrial membrane potential, intracellular NAD levels, and the NAD⁺/NADH ratio were all significantly elevated (Fig. 6F-H), suggesting enhanced mitochondrial function and oxidative phosphorylation efficiency.

Together, these findings indicate that the OBCF strategy promotes a metabolic shift toward more efficient energy production during the LSP, thereby supporting sustained antibody productivity under nutrient-rich yet metabolically stressful conditions.

**Fig. 7:**
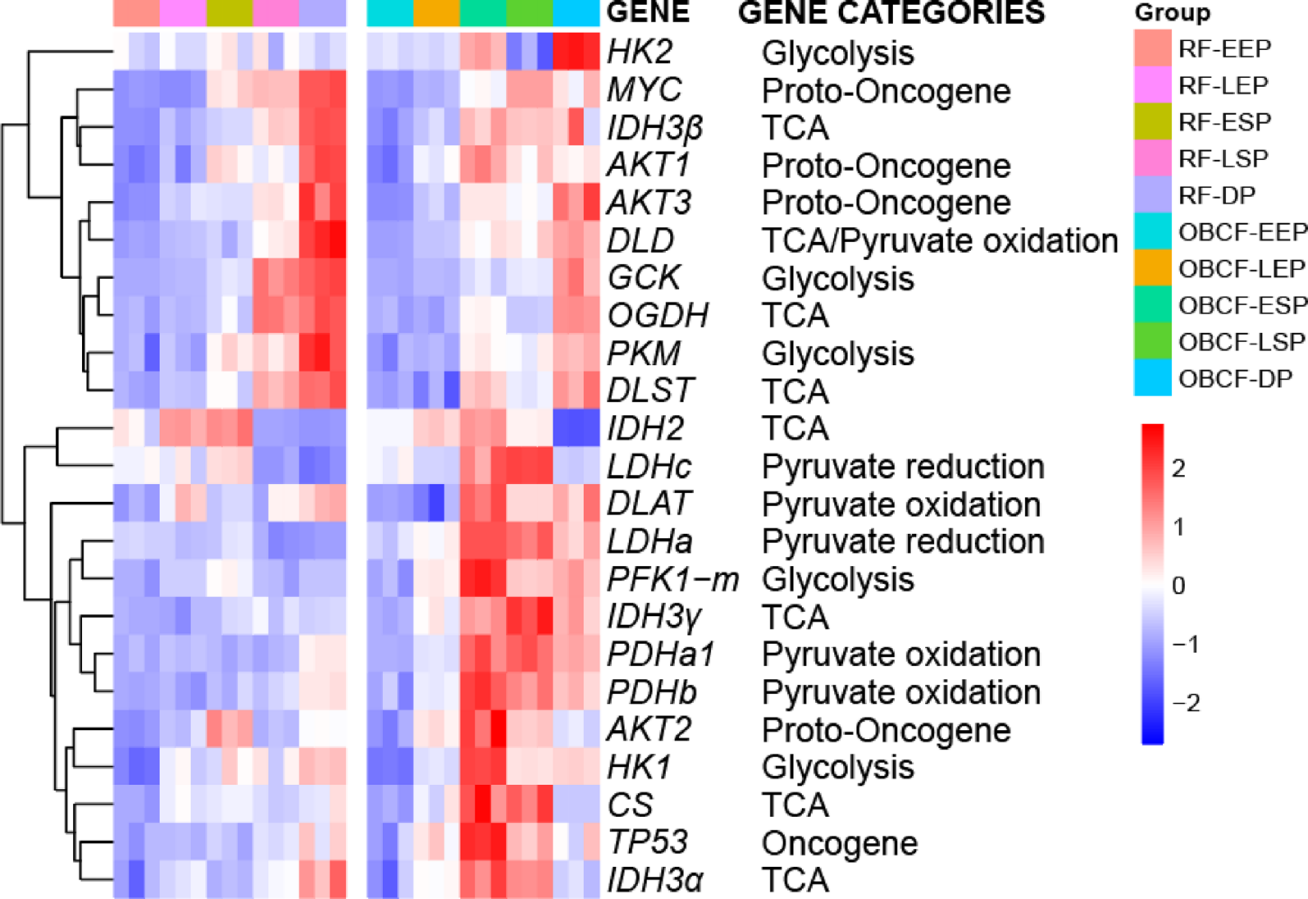
Heatmap of mRNA levels of key enzymes in central carbon metabolism during the five phases of fed-batch culture under the RF and OBCF strategies. Data from three biological replicates were normalized to the EEP. Each row represents a key enzyme, while each column corresponds to a specific culture phase. Cluster analysis grouped the genes into two distinct expression patterns, reflecting different regulatory responses to metabolic demands under the RF and OBCF strategies

To elucidate the transcriptional underpinnings of the metabolic reprogramming observed under the OBCF strategy, mRNA expression levels of key genes involved in central carbon metabolism across five fed-batch phases were quantified (Fig. 7). Unsupervised clustering separated genes into two response cohorts. Under the reference feeding (RF) strategy, transcripts associated with oncogenic signaling (*MYC*, *AKT1/3*), glycolysis (*GCK*, *PKM*), and the TCA cycle (*OGDH*, *DLD*, *DLST*) exhibited progressive upregulation, peaking in the decline phase. In contrast, OBCF markedly attenuated this late‐stage induction. Conversely, OBCF selectively enhanced early and sustained expression of genes driving flux into the TCA cycle, including pyruvate dehydrogenase subunits (*PDHa1*, *PDHb*, *DLAT*), TCA rate-limiting genes (*CS*, *IDH2*, *IDH3γ*), lactate oxidation-associated genes (*LDHA*, *LDHC*), starting from the early stationary phase.

**Table 1:** Differential transcriptional levels of key enzymes during the late stationary phase (LSP) under RF and OBCF strategies. Bold font indicates genes with |Fold change| ≥ 1.5 and p < 0.05 (statistically significant)

Categories	Gene	RF-LSP	OBCF-LSP	Fold Change	*p*-value
Glycolysis	*HK1*	1.61 ± 0.17	1.78 ± 0.01	1.10	0.213
	***HK2***	**1.05 ± 0.18**	**0.66 ± 0.14**	**0.63**	0.045*
	***GCK***	**25.22 ± 1.52**	**8.42 ± 0.11**	**0.33**	< 0.001***
	***PFK1-m***	**1.07 ± 0.11**	**1.97 ± 0.01**	**1.84**	0.002**
	*PKM*	1.68 ± 0.08	1.47 ± 0.08	0.87	0.105
Lactate	***LDHA***	**0.77 ± 0.06**	**1.73 ± 0.07**	**2.25**	0.008**
Metabolism	***LDHC***	**0.72 ± 0.03**	**1.58 ± 0.01**	**2.19**	< 0.001***
Pyruvate	***PDHα1***	**1.03 ± 0.06**	**2.49 ± 0.10**	**2.42**	< 0.001***
Dehydrogenase	***PDHβ***	**1.23 ± 0.07**	**2.09 ± 0.10**	**1.70**	0.013*
Complex	*DLAT*	1.16 ± 0.11	1.29 ± 0.00	1.11	0.187
	*DLD*	2.19 ± 0.12	2.28 ± 0.24	1.04	0.654
TCA	***CS***	**1.19 ± 0.04**	**2.01 ± 0.13**	**1.69**	0.009**
	***IDH2***	**0.53 ± 0.00**	**1.13 ± 0.02**	**2.13**	0.004**
	*IDH3α*	1.31 ± 0.02	1.72 ± 0.02	1.31	0.022*
	*IDH3β*	2.29 ± 0.12	2.42 ± 0.02	1.06	0.354
	***IDH3γ***	**1.24 ± 0.12**	**2.40 ± 0.11**	**1.94**	0.017*
	***OGDH***	**2.47 ± 0.11**	**1.22 ± 0.01**	**0.49**	0.001**
	*DLST*	2.22 ± 0.08	1.51 ± 0.05	0.68	0.003**
Regulatory Pathways	*TP53*	1.33 ± 0.04	1.90 ± 0.10	1.43	0.037*
	*MYC*	3.24 ± 0.03	3.62 ± 0.01	1.12	0.042*
	*AKT1*	1.31 ± 0.03	1.42 ± 0.07	1.08	0.289
	*AKT2*	1.18 ± 0.04	1.75 ± 0.02	1.48	0.021*
	*AKT3*	2.20 ± 0.09	1.95 ± 0.04	0.89	0.156

Differential expression analysis during the late stationary phase (Table 1) highlighted key regulatory shifts. In the glycolytic pathway, transcript levels of *PFK1-m* were upregulated under OBCF, while upstream regulators such as *GCK* and *HK2* were suppressed, indicating a possible compensatory mechanism to maintain glycolytic flux. In pyruvate metabolism, elevated mRNA levels of *PDHa1* and *PDHb* suggested enhanced pyruvate entry into the TCA cycle. Although *LDHA* and *LDHC* transcripts were both upregulated, the altered subunit ratio may favor lactate oxidation over production, consistent with the observed reduction in J_PYR−LAC_ flux.

Within the TCA cycle, increased transcript levels of *CS*, *IDH2*, and *IDH3γ* under OBCF parallels elevated flux through rate-limiting steps. Notably, despite transcriptional downregulation of *OGDH* and *DLST*, corresponding fluxes remained elevated, implying possible post-transcriptional compensation.

Collectively, these transcriptional adaptations under OBCF align with observed flux redistribution, dampened glycolytic overflow and bolstered oxidative metabolism, thereby underpinning sustained ATP generation and improved antibody productivity during the late stationary phase.

## Discussion

### Environmental drivers of Q_P_ decline under RF strategy

This study identified that the imbalanced amino acid supply under the RF strategy led to a 99 mM surplus, elevating osmolarity to 536 mOsm/kg by day13. While moderate hyperosmolarity (e.g., 450 mOsm/kg) has been reported to enhance antibody production (Nasseri et al. 2014), excessive osmolarity (> 530 mOsm/kg) has detrimental effects, including cell cycle arrest, mitochondrial DNA damage, and mitochondrial membrane depolarization Romanova et al. (2022). These observations suggest that the amino acid overaccumulation and the associated osmolarity increase were key factors contributing to the Q_P_ decline during the LSP. In contrast, fed-batch cultures employing the OBCF strategy effectively mitigated this accumulation, delaying osmolarity rise above 530 mOsm/kg until day 16, thereby reducing metabolic stress and supporting sustained productivity.

Beyond osmolality, byproducts beyond lactate and ammonia may also contribute to LSP-associated productivity loss. CHO cells are known to secrete various branched-chain amino acid during fed-batch cultures (Bethune et al. 2021; Mulukutla et al. 2017), which may impact cell growth and antibody production. Notably, Mulukutla et al. (2017) demonstrated that controlling the concentrations of specific amino acids (Phe, Tyr, Trp, Met, Leu, Ser, Thr, Gly) within 0.5-1 mmol/L reduces inhibitory metabolite buildup and support higher cell densities and increased productivity. The OBCF strategy reduced excessive amino acid levels, potentially limiting these toxic byproducts and contributing to improved yield and batch consistency.

In this study, only extracellular amino acids were quantified. Their continuous accumulation suggested that intracellular pools were likely sufficient, and these measurements were adequate for MFA. Future studies combining extracellular and intracellular profiling could provide a more comprehensive understanding of metabolic regulation and guide process optimization.

### Design and dynamic control logic of the OBCF strategy

Unlike static equal-volume feeding strategies such as RF that are unable to accommodate dynamic metabolic changes, OBCF leverages real-time OUR signals to achieve precise, demand-driven nutrient modulation throughout the culture process.

Compared to previous OUR-based dynamic feeding strategies, this study introduces significant advancements in control logic. While earlier studies have successfully utilized OUR to regulate the supply of key nutrients such as glucose (Casablancas et al. 2013) or both glucose and glutamine (Martínez-Monge et al. 2022). Expanding upon this concept, our strategy utilizes OUR-derived oxygen uptake data to infer the overall amino acid uptake rate (AUR), enabling real-time adjustment of concentrated medium feeding. This holistic approach addresses the non-constant nature of amino acid demand during fed-batch processes, allowing dynamic supply optimization across multiple amino acids. As a result, the OBCF strategy ensures a better match between nutrient supply and cellular demand, mitigates osmotic stress caused by nutrient imbalance, and ultimately enhances antibody productivity and process stability. It should be noted that the quantitative relationship between OUR and AUR may vary across different cell lines or expressed proteins, and may need to be determined empirically for new systems.

Looking forward, integrating OUR-based amino acid regulation with existing glucose/glutamine feedback control strategies may enable multi-nutrient, real-time optimization of fed-batch processes. Such integration could further enhance process robustness and production efficiency in industrial antibody manufacturing.

### Mechanistic insights into productivity enhancement under OBCF

Antibody production requires substantial energy input and sustained oxidative metabolism (Templeton et al. 2013). Mitochondrial membrane potential and redox indicators, such as total NAD and the NAD⁺/NADH ratio, are widely used to assess energy metabolism in CHO cells (Viña et al. 2016; Zorova et al. 2018). In this study, the OBCF strategy significantly enhanced these energy-related indicators during the late stationary phase (LSP), coinciding with increased antibody-specific productivity (Q_P_), suggesting that improved energy metabolism plays a pivotal role in sustaining production at this stage.

Transcriptomic profiling further revealed distinct expression dynamics of central carbon metabolism genes across culture phases. Under the RF strategy, key genes related to glycolysis and the TCA cycle (e.g., *MYC*, *PKM*, *OGDH*) were upregulated primarily during the decline phase, likely reflecting a delayed cellular response to energy stress. In contrast, OBCF induced earlier transcriptional activation—beginning in the early stationary phase—of genes supporting pyruvate oxidation and TCA flux, including *PDHa1*, *PDHb*, *DLAT*, *CS*, and *IDH3γ*. This anticipatory gene regulation may help redistribute metabolic flux toward oxidative phosphorylation, ensuring timely energy supply for biosynthetic demands during the LSP.

Although this study highlights the role of central carbon metabolism, a comprehensive understanding of OBCF’s regulatory impact will require integrated transcriptomic, proteomic, and functional analyses, given the complexity of energy-related pathways and the limitations of mRNA-level data alone.

Previous studies have shown that targeted regulation of metabolic enzymes can improve CHO cell productivity. For example, *LDHA* knockout has been reported to prevent lactate accumulation and increase Fc-fusion protein yield (Wilkens et al. 2019), while overexpression of pyruvate carboxylase (*PYC2*) enhances TCA flux and reduces lactate production (Gupta et al. 2017; Kim and Lee 2007). However, few studies have explored the overexpression of TCA branchpoint enzymes such as *CS*, *IDH3*γ, or *OGDH*. Future efforts may focus on synergistic engineering of these targets or the use of inducible systems to modulate expression in a stage-specific manner, further improving energy metabolism and product yields during the LSP.

## Conclusion

This study systematically investigated the decline in specific antibody productivity (Q_P_) during the late stationary phase (LSP) of fed-batch CHO cell cultures and proposed a novel oxygen uptake rate OUR based continuous feeding (OBCF) strategy to address this challenge. Under the conventional reference feeding (RF) strategy, excessive amino acid accumulation led to elevated osmolality and potential buildup of inhibitory byproducts, contributing to cellular metabolic reprogramming, mitochondrial dysfunction, and compromised energy metabolism—culminating in reduced Q_P_. By leveraging OUR as a real-time indicator of cellular metabolic demand, the OBCF strategy enabled dynamic adjustment of feeding volume, thereby minimizing osmotic stress and aligning nutrient supply with consumption. Compared to RF, the OBCF strategy improved metabolic efficiency, enhanced mitochondrial function, and sustained oxidative phosphorylation during the LSP. These improvements resulted in a 52% increase in Q_P_ during the LSP and a 32% higher final antibody titer. Collectively, our findings underscore the importance of dynamic, metabolism-guided nutrient control for maintaining high cellular productivity in prolonged cultures. The OBCF strategy offers a scalable and versatile framework for real-time process optimization, with potential applications across diverse biopharmaceutical production platforms.

## Supplementary information

Below is the link to the electronic supplementary material.

Supplementary Material 1

## Data Availability

Data will be made available on reasonable request.

## Abbreviations

**Q**_**P**_: Specific Antibody Production Rate (pg/cell/day)
**OUR**: Oxygen Uptake Rate (mmol/L/h)
**VCD**: Viable Cell Density (10^9^ cell/L)
**EEP**: Early exponential phase
**LEP**: Late exponential phase
**ESP**: Early stationary phase
**LSP**: Late stationary phase
**DP**: Decline phase
**μ**: Specific growth rate (day⁻^1^)
**Q**_**AA**_: Specific amino acid consumption rate (mmol/10^9^ cells/day)
**AUR**: Amino acid uptake rate (mmol/L/h)
**Q**_**ATP**_: Specific ATP production rate (mmol/10^9^ cells/h)
**CHO**: Chinese hamster ovary
**OBCF**: Strategy OUR-based continuous feeding (OBCF) strategy
**NAD**^**+**^: Nicotinamide Adenine Dinucleotide (oxidized form)
**NADH**: Nicotinamide Adenine Dinucleotide (reduced form)
