# Supplementary material for: Integrating real-time OUR monitoring with adaptive feeding for enhanced antibody production
Source: Bioresour Bioprocess. 2025 Dec 20;12(1):150. doi: 10.1186/s40643-025-00961-x (PMC12718288; doi:10.1186/s40643-025-00961-x)
Supplement: Supplementary file 1 — Supplementary Material 1 [file 40643_2025_961_MOESM1_ESM.docx]

# Supplementary Materials

## Figure S1


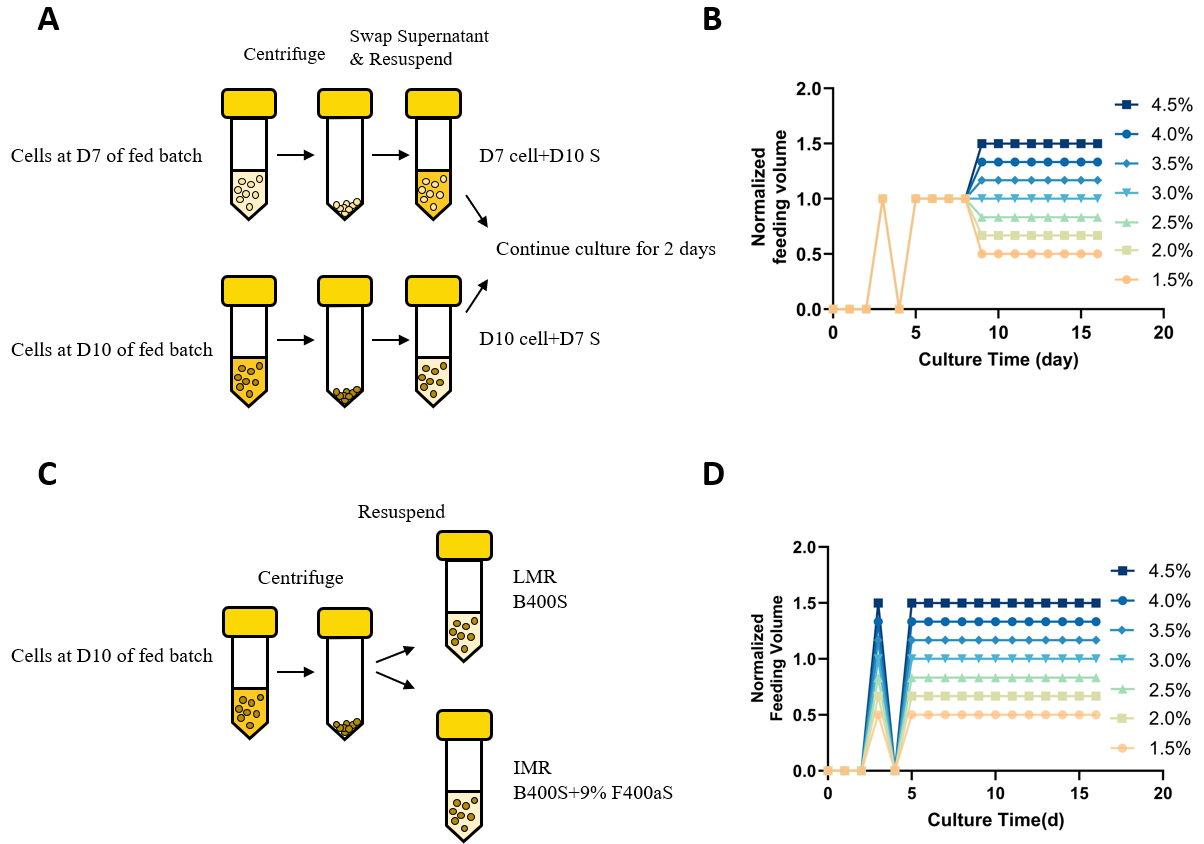


**Figure S1** Schematic diagrams of the experimental setups in Section 2.2.4: (A) Supernatant Exchange Test (operation schematic), (B) Dose-response to feeding volume (Late Course; feeding profile), (C) Medium Replacement Test (operation schematic). (D) Dose-response to feeding volume (Full Course; feeding profile).

## Figure S2


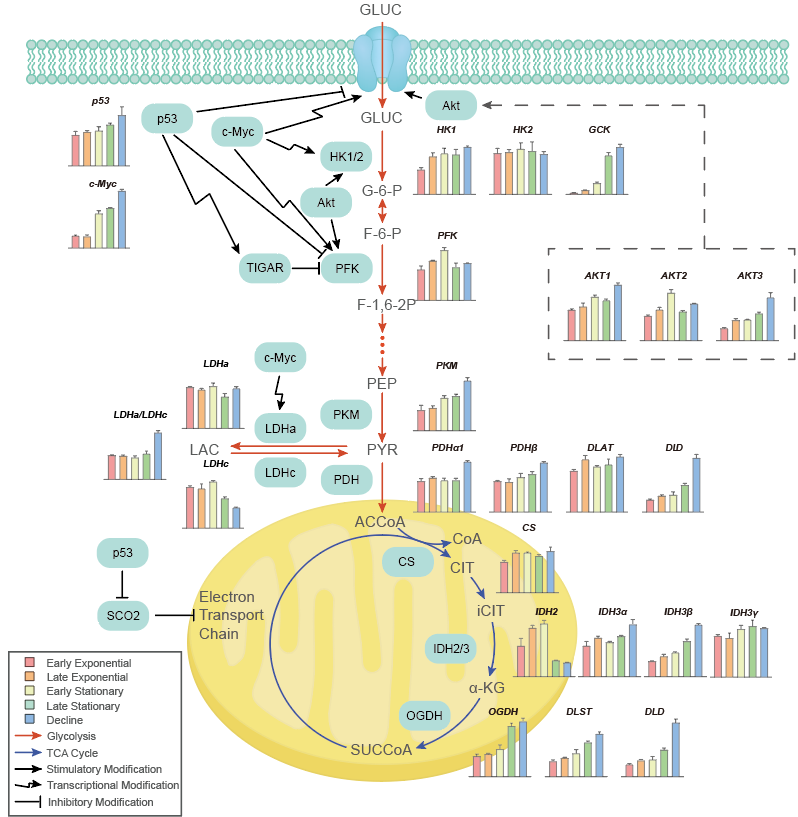


**Figure S2** Catalytic sites and transcriptional levels of key enzymes of central carbon metabolism in CHO fed-batch culture with the RF strategy.

The bar chart represents the transcriptional levels of key enzymes in central carbon metabolism across five phases. Red represents EEP, orange represents LEP, yellow represents ESP, green represents LSP, and blue represents DP. The sky-blue rounded rectangles highlight the key enzymes in central carbon metabolism. Red arrows indicate glycolysis, blue arrows indicate the TCA cycle, and the black lines represent the modes of enzyme action: stimulatory modification, transcriptional modification, and inhibitory modification.

It should be noted that while OGDH transcription increased from ESP to LSP, the flux from α-KG to SUCCoA (J_AKG-SUC_, Fig. 2E) decreased. This apparent discrepancy likely reflects the complex regulation of metabolic flux: enzyme abundance alone does not dictate flux, which is also influenced by substrate availability, cofactor levels, and alternative carbon flux distributions. Such decoupling between transcriptional levels and metabolic flux is commonly observed in central carbon metabolism and highlights the dynamic regulation under different culture stages.

## Figure S3

**Figure S3** Relative mRNA level of (a) *LDHA/LDHC* (b) *IDH2* in ESP and LSP with the RF strategy.

As shown in Figure S2A, the ratio of *LDHA/LDHC* in the LSP is significantly higher than in the ESP. In the interconversion between pyruvate and lactate, LDHA catalyzes the conversion of pyruvate to lactate, while LDHC catalyzes the conversion of lactate to pyruvate (as shown in Figure S1). The increase in the *LDHA/LDHC* ratio may be responsible for the greater pyruvate flux towards lactate production rather than entering the TCA cycle in the LSP.

As shown in Figure S2B, the transcriptional level of *IDH2* in the LSP is significantly lower than in the ESP. The reduction in *IDH2* transcription may lead to a decrease in the TCA cycle flux.

## Figure S4

**Figure S4** (A) Q_P_ of ESP and LSP after medium replacement via centrifugation. (B) Comparison of antibody-specific productivity between LMR and IMR five days post medium replacement.

As shown in Figure S3(A), the control group exhibited a significant decline in Q_P_ during the LSP. In contrast, both the LMR and IMR groups maintained stable Q_P_ levels after medium replacement, indicating that centrifugation and medium replacement effectively alleviated the decline in Q_P_ during LSP. Notably, the stability observed in IMR suggests that factors beyond osmolarity might also contribute to the Q_P_ decline.

Figure S3(B) shows that five days post medium replacement, Q_P_ in the IMR group was significantly lower than in the LMR group. This result reinforces the conclusion that osmolarity indeed plays a critical role in Q_P_ decline during the LSP.

## Figure S5


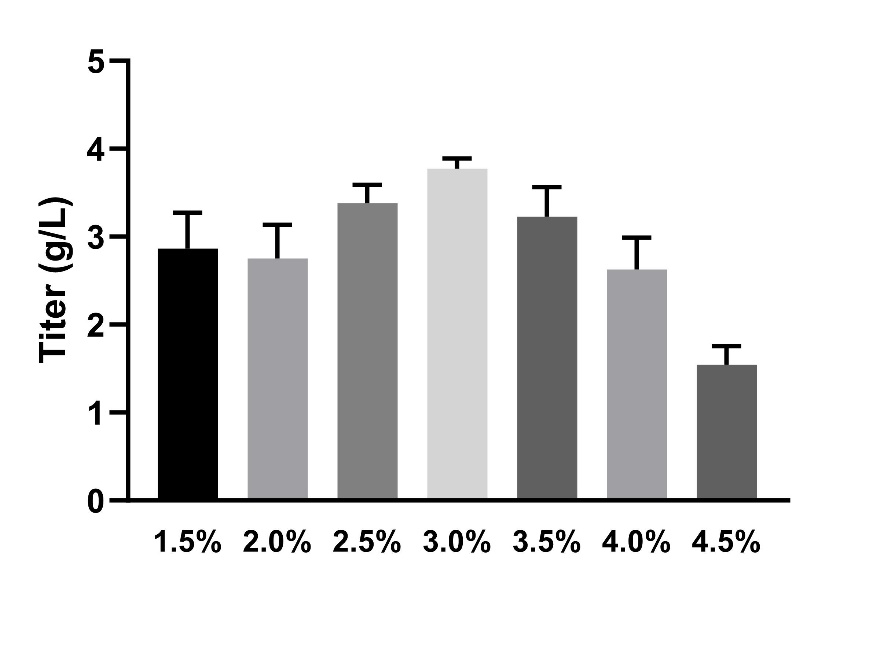


**Figure S5** Relationship between feeding amount and productivity in the equimolar bolus feeding strategy. 3.0% represents RF strategy.

## Figure S6


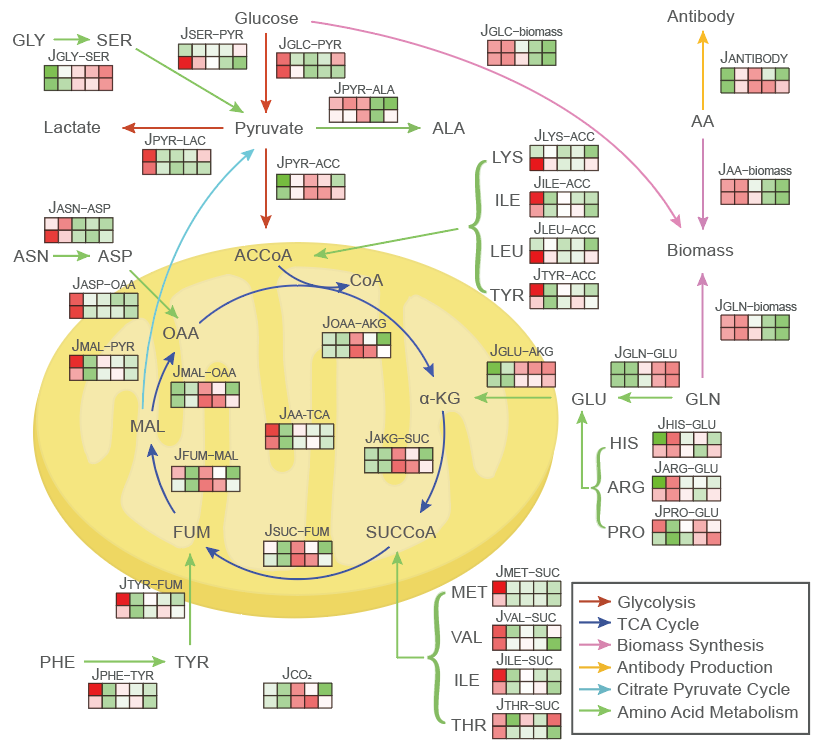


**Figure S6** Global overview of metabolic fluxes across five culture phases under RF and OBCF strategies

A 5×2 grid represents each pathway: the first row corresponds to the five phases (EEP, LEP, ESP, LSP, DP) under the RF strategy, and the second row represents the corresponding five phases under the OBCF strategy. The color gradient indicates metabolic flux levels, with red representing higher fluxes and green representing lower fluxes. Comparisons highlight differences in glycolysis, TCA cycle activity, amino acid metabolism, and other pathways between the two strategies.

## Table S1

**Table S1** Bioreactions in the simplified metabolic network of GS-CHO cells

|  | Fluxs | | Reactions | |  |
| --- | --- | --- | --- | --- | --- |
| Glycolysis: | J_GLC-PYR_ | | GLC → 2PYR | |  |
|  | J_PYR-LAC_ | | PYR → LAC | |  |
| TCA cycle: | J_PYR-ACC_ | | PYR → AcCoA+CO_2_ | |  |
|  | J_PYR-AKG_ | | AcCoA+OAA → α-KG+CO_2_ | |  |
|  | J_AKG-SUC_ | | α-KG → SucCoA+CO_2_ | |  |
|  | J_SUC-FUM_ | | SucCoA→FUM | |  |
|  | J_FUM-MAL_ | | FUM → MAL | |  |
|  | J_MAL-OAA_ | | MAL → OAA | |  |
| Biomass Synthesis: | J_GLC-Biomass_ | | α_1_GLC+α_2_GLN+α_i_Amino Acids → Cells | |  |
| Antibody production: | J_mAb_ | | β_1_GLN+β_i_Amino Acids → Antibody | |  |
| Citrate Pyruvate Cycle: | | J_MAL-PYR_ | | MAL → PYR+CO_2_ | |
| Amino Acid Metabolism: | J_PYR-ALA_ | | PYR+GLU → ALA+α-KG | |  |
|  | J_GLN-GLU_ | | GLN → GLU+NH_3_ | |  |
|  | J_GLU-AKG_ | | GLN → α-KG+NH_3_ | |  |
|  | J_SER-PYR_ | | SER → PYR +NH_3_ | |  |
|  | J_GLY-SER_ | | 2GLY → SER+CO2+NH_3_ | |  |
|  | J_CYS-PYR_ | | CYS → PYR+NH_3_ | |  |
|  | J_ASP-OAA_ | | ASP+α-KG → OAA+GLU | |  |
|  | J_ASN-ASP_ | | ASN → ASP+CO_2_ | |  |
|  | J_HIS-GLU_ | | HIS → GLU+2NH_3_+CO_2_ | |  |
|  | J_ARG-GLU_ | | ARG+α-KG → 2GLU+2NH_3_+CO_2_ | |  |
|  | J_PRO-GLU_ | | PRO → GLU | |  |
|  | J_ILE-SUC_ | | ILE +α-KG → SucCoA+AcCoA+GLU | |  |
|  | J_VAL-SUC_ | | VAL +α-KG → GLU+CO_2_+SucCoA | |  |
|  | J_MET-SUC_ | | MET+SER +α-KG → CYS+SucCoA+GLU | |  |
|  | J_THR-SUC_ | | THR → SucCoA+NH_3_ | |  |
|  | J_PHE-TYR_ | | PHE → TYR | |  |
|  | J_TYR-FUM_ | | TYR+α-KG → GLU+FUM+2AcCoA+CO_2_ | |  |
|  | J_LYS-ACC_ | | LYS+2α-KG → 2GLU+2CO_2_+2AcCoA | |  |
|  | J_LEU-ACC_ | | LEU+α-KG → GLU+3AcCoA | |  |

## Table S2

**Table S2** Primer sequences for qPCR

| Gene | Forward Primer (5' - 3') | Reverse Primer (3' - 5') |
| --- | --- | --- |
| *HK1* | CGGCTCAGAAAAGGGGGATT | GATGTTCTCCGGGGTTTCGT |
| *HK2* | GGCAGTGGAACCCAGCTATT | ATGTCAAAGTCCCCTCTGCG |
| *GCK* | CCGCAAGCAAATCCACAACA | GCGATTTATGACCCCGGCTA |
| *PFK1-m* | CCGTGGTTCTCGTCTCAACA | TCAAAGGCTGATGGTGTCCC |
| *PKM* | TGGATCTCCCTGCTGTGTCA | CATCTTCTGCCTTGCGGATG |
| *OGDH* | GGAGCTGGTAACAAACCGGA | CTCAAAGCCCAGGACACCAT |
| *DLST* | CGGCTGTGTGCAAGAATGAAG | CAACTTTGCCCCCATCAGGTA |
| *DLAT* | CCTCTCCCCCACCATGACTA | GTCACTTCCGTTGGCCTGTA |
| *CS* | TGCACCCCATGTCTCAACTC | TGGGACCAGTCCAGCTTAGA |
| *DLD* | CGCATACTACAAAAGCAAGGCT | TCGACCGATGCAAACCAAGA |
| *PDHα1* | ATTGCTCTGGCCTGCAAGTA | GCCTCTCGGACACACAAGAT |
| *PDHβ* | TCTGGACTTGTGAAGCGGC | TTCCAGCAAAGCCCATCTCT |
| *LDHA* | CCTCAGTGTCCCTTGTGTCC | TGCACTCTTCTTCAAGCGGG |
| *LDHC* | GCTTAGGACAAGAATTGGGGACA | CTCCAAAAGAATGGCCACAGC |
| *AKT1* | GTGAGCGTGTGTTTTCCGAG | CCAGGTACTCAGGTGTTCCG |
| *AKT2* | TGTCGCAGAATGCCAACTGA | AAGGCATTGTCACCTGGGTC |
| *AKT3* | GGCCTGGGTGTTGTCATGTA | CCCTCCACCAAGGCGTTTAT |
| *TP53* | GTTTGGCTCCTCCTCAGCAT | CTCCCAGGACAGGCACAAAT |
| *c-MYC* | GATGCCACGTCTCCACTCAT | CAACTCCGGGATCTGGTCAC |
| *IDH2* | GGAGATGGACGGCGATGAGA | TGAAGACGGTTCCCCCAAGGA |
| *IDH3α* | TGGAACAGCCCCAGACATTG | CGCAGTCTCGATTTTGGCTG |
| *IDH3β* | CCTGGTGAGAGCTACAGTGC | GAAGCTGACAACAGCATGGC |
| *IDH3γ* | GGTTGGAGGGTCAGGACTTG | TCTGGTGTGCAGATGGCTTT |
| *β-actin* | CCCCATTGAACACGGCATTG | TCTTTTCACGGTTGGCCTTG |

## Table S3

**Table S3**. Intracellular Metabolic Fluxes during ESP and LSP under the RF Strategy

| Fluxes | RF-ESP | RF-LSP |
| --- | --- | --- |
| J_GLC-PYR_ | 0.302±0.008 | 0.330±0.011 |
| J_PYR-LAC_ | -0.001±0.006 | **0.068±0.008^*^** |
| J_PYR-ACC_ | 0.181±0.001 | **0.153±0.002^***^** |
| J_OAA-AKG_ | 0.440±0.008 | **0.343±0.027^**^** |
| J_AKG-SUC_ | 0.328±0.002 | **0.253±0.017^**^** |
| J_SUC-FUM_ | 0.323±0.001 | **0.238±0.020^*^** |
| J_FUM-MAL_ | 0.307±0.001 | **0.219±0.025^*^** |
| J_MAL-OAA_ | 0.335±0.005 | 0.271±0.019 |
| J_GLN-GLU_ | -0.033±0.002 | **-0.021±0.001^**^** |
| J_GLU-AKG_ | -0.024±0.005 | -0.019±0.009 |
| J_MAL-PYR_ | -0.025±0.003 | -0.042±0.006 |
| J_PYR-ALA_ | 0.013±0.000 | 0.004±0.002 |
| J_SER-PYR_ | 0.006±0.001 | 0.008±0.003 |
| J_GLY-SER_ | -0.004±0.000 | -0.004±0.000 |
| J_CYS-PYR_ | 0.002±0.001 | 0.009±0.008 |
| J_ASP-OAA_ | 0.021±0.001 | **0.008±0.002^*^** |
| J_ASN-ASP_ | 0.023±0.001 | 0.025±0.002 |
| J_HIS-GLU_ | 0.004±0.001 | 0.005±0.003 |
| J_ARG-GLU_ | 0.006±0.002 | 0.004±0.006 |
| J_PRO-GLU_ | -0.014±0.000 | **-0.010±0.000^****^** |
| J_ILE-SUC_ | -0.002±0.000 | **-0.004±0.001^*^** |
| J_VAL-SUC_ | 0.002±0.000 | **-0.001±0.001^*^** |
| J_MET-SUC_ | -0.001±0.000 | -0.001±0.000 |
| J_THR-SUC_ | -0.000±0.001 | -0.003±0.001 |
| J_PHE-TYR_ | -0.011±0.000 | -0.014±0.006 |
| J_TYR-FUM_ | -0.012±0.000 | -0.014±0.004 |
| J_LYS-ACC_ | -0.001±0.000 | -0.001±0.001 |
| J_LEU-ACC_ | -0.001±0.000 | -0.001±0.001 |
| J_BIOMASS_ | 0.155±0.018 | **0.100±0.000^*^** |
| J_ANTIBODY_ | 0.058±0.006 | **0.027±0.004^**^** |
